# Fluorescent Waveguide Lattices for Enhanced Light Harvesting and Solar Cell Performance

**DOI:** 10.1021/acsaem.3c00687

**Published:** 2023-06-09

**Authors:** Nannan Ding, Ian D. Hosein

**Affiliations:** Department of Biomedical and Chemical Engineering, Syracuse University, Syracuse, New York 13244, United States

**Keywords:** solar cells, polymer blends, coatings, energy conversion, waveguides

## Abstract

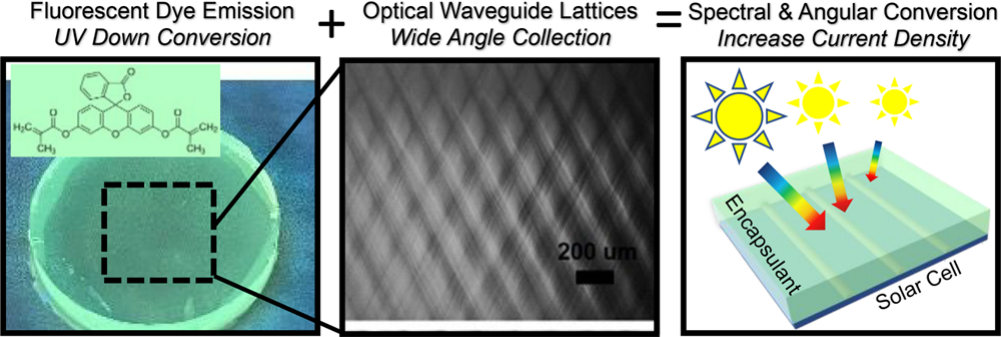

We present the properties and performance of fluorescent waveguide lattices as coatings for solar cells, designed to address the significant mismatch between the solar cell’s spectral response range and the solar spectrum. Using arrays of microscale visible light optical beams transmitted through photoreactive polymer resins comprising acrylate and silicone monomers and fluorescein *o*,*o*′-dimethacrylate comonomer, we photopolymerize well-structured films with single and multiple waveguide lattices. The materials exhibited bright green-yellow fluorescence emission through down-conversion of blue-UV excitation and light redirection from the dye emission and waveguide lattice structure. This enables the films to collect a broader spectrum of light, spanning UV–vis–NIR over an exceptionally wide angular range of ±70°. When employed as encapsulant coatings on commercial silicon solar cells, the polymer waveguide lattices exhibited significant enhancements in solar cell current density. Below 400 nm, the primary mode of enhancement is through down-conversion and light redirection from the dye emission and collection by the waveguides. Above 400 nm, the primary modes of enhancement were a combination of down-conversion, wide-angle light collection, and light redirection from the dye emission and collection by the waveguides. Waveguide lattices with higher dye concentrations produced more well-defined structures better suited for current generation in encapsulated solar cells. Under standard AM 1.5 G irradiation, we observed nominal average current density increases of 0.7 and 1.87 mA/cm^2^ for single waveguide lattices and two intersecting lattices, respectively, across the full ±70° range and reveal optimal dye concentrations and suitable lattice structures for solar cell performance. Our findings demonstrate the significant potential of incorporating down-converting fluorescent dyes in polymer waveguide lattices for improving the current spectral and angular response of solar cell technologies toward increasing clean energy in the energy grid.

## Introduction

Solar energy is becoming increasingly important as a source of clean and renewable energy. According to the International Energy Agency, global solar PV capacity has grown from 8 GW in 2009 to over 770 GW in 2020, representing a significant increase in solar energy adoption worldwide. Solar energy has the potential to reduce greenhouse gas emissions and provide access to electricity in remote and off-grid areas. Therefore, developing more efficient and cost-effective solar energy technologies is crucial to meeting the world’s growing energy demand while reducing our dependence on fossil fuels. In addition to land-based applications, solar cells are also critical for space technologies such as spacecraft and satellites. Solar cells must be able to operate in extreme environments, collecting as much solar radiation to maximize power deliver to space technologies. Hence, development of more efficient and reliable solar cells can greatly improve the performance and longevity of space technologies, enabling a wide range of applications from communications and navigation to weather monitoring and scientific research. Thus, solar cell research and development is important not only for Earth-based energy solutions but also for advancements in space technology.

To improve conversion and electrical output, researchers are exploring various light management techniques to increase total energy capture, mitigate shading losses, and improve conversion efficiencies. While techniques can be implemented at either the module or fundamental cell level, using advanced coatings as the encapsulation layer over the solar cell offers an attractive alternative because it can easily integrate into existing silicon solar technology and may even eliminate the need for complex module architectures or solar tracking methods. Coatings also offer opportunities to leverage the structure–property relations of a wide range of optical surface structures. To date, improvements in solar cell performance have been demonstrated through nanoparticles surface coatings, nanostructured diffraction and diffuse layers, nanotexturing, geometric optical structure, among many others.^1−19^

Multiwaveguide lattices (MWGLs) have recently gained attention as an effective means of controlling the collection and transmission of light when employed as encapsulants for solar cells.^20,21^ The waveguide lattices are synthetically organized in photoreactive polymer blends, consisting of a high refractive index acrylate-based free-radical polymerizing resin and a low refractive index cationic polymerizing epoxide-terminated silicone resin.^22^ The mixed resin is irradiated by arrays of microscale optical beams that are transmitted through the medium. Each optical beam undergoes self-focusing in the photoreactive medium’s nonlinearity, resulting in self-trapped optical beams propagating divergence-free.^23^ These self-trapped optical beams each inscribe optically a waveguide of cylindrical geometry along their path of propagation, thereby forming arrays of waveguides (i.e., a lattice). With continued increase in molecular weight during photopolymerization, the cylindrical regions undergo polymerization induced phase separation (PIPS),^20,21^ expelling the low refractive index silicone polymer into the surrounding regions, thereby producing the high-index core and low-index cladding geometry that are essentially step index microscale optical fibers.

Advantageously, by synthesizing MWGLs comprising even just two lattices each at slanted orientations relative to the surface normal (e.g., ±30°), their acceptance ranges could be angularly rotated to collect more light at more glancing incident angles. Thereby, wide-angle incident radiation is collected and transmitted across the polymer through the waveguides at sharper angles (i.e., closer to the surface normal) than otherwise dictated by Snell’s law.^20,21^ The combination of multiple (two is in fact sufficient) intersecting waveguide lattices can sweep the entire practical collection window (−70 to 70°) enabling the entire angular range to be efficiently collected and directed toward the solar cell, as compared to both a uniform film and single vertically aligned lattice. Transmitting ultrawide angles of light via more direct paths toward the solar cell mitigates effects that cause losses, such as shading from the solar cell’s front contacts,^13,24,25^ thereby leading to efficiency enhancements. This wide-angle light conversion would allow greater flexibility in solar installation locations and orientations and extends time of sustained energy generation, e.g., earlier into morning, later into evening, and in winter seasons (i.e., sun is closer to the horizon). The photovoltaic modules are more *omnidirectional*, i.e., position/orientation agnostic, and thus versatile in powering infrastructure, homes, buildings, and nonoptimal geographic locations.

Yet another critical issue that persists with photovoltaic technology is the significant mismatch between their spectral response and the solar radiation spectrum, which limits their ability to collect light over a wider range of wavelengths. Silicon (Si) solar cells, the dominant type of solar cell for energy conversion on Earth and in space technologies, have a narrow spectral response window (∼400–900 nm) with sharp drop-offs in the UV and IR regions, leaving a significant portion of the solar radiation unharvested. This is particularly important for space technologies such as satellites and spacecraft, which rely on solar cells to generate electricity from sunlight. A significant portion of the sun’s energy is in the form of UV radiation, with UV-A radiation (wavelengths between 315 and 400 nm) being the most abundant in the solar spectrum. However, most solar cells are not efficiently able to convert UV radiation into electricity. Therefore, the collection of UV radiation is especially important for both land and space applications of solar cells. Creating a polymer coating that can convert UV-A radiation into visible light can increase the amount of solar energy harvested by solar cells, potentially leading to more efficient and reliable space technologies. This technology could also have significant benefits for purely land-based applications, such as residential and commercial installations, solar farms, and other renewable energy projects. By enabling solar cells to collect more energy from the UV portion of the solar spectrum, this technology could increase the efficiency and overall energy output of solar panels. This would lead to lower costs and greater adoption of solar energy, which could help to reduce dependence on fossil fuels and mitigate the impacts of climate change.

Fluorescent dye incorporation into solar cell architectures is a well-known approach to increase the conversion of solar radiation from the UV regime, specifically through down-conversion of high energy UV photons into the visible regime. This has been pursued both to increase total solar energy conversion and to provide UV protection of the cell and degradation resistance of the encapsulant itself.^26,27^ However, up until now, materials exploration of down conversion particularly in solar cell encapsulation materials have focused primarily on rare-earth-doped crystalline materials and quantum dots.^26^ Dye-incorporated polymer encapsulants are a relatively unexplored area in encapsulation technology. Only one example exists, in which an EVA layer over a CdTe cell included a perylene- or naphtalimide-based fluorophore dye,^28^ for which, depending on the dye, short circuit current densities improve by 4–9%. When incorporated in an acrylate-based resin (e.g., PMMA), fluorescent dyes can show excellent stability under 1 sun conditions for >5000 h.

In this work, we report on the synthesis of waveguide lattices for use as coatings (i.e., encapsulants) for Si solar cells. We formulated polymer blends with a dye-tagged acrylate comonomer and investigated the formation of single waveguide and two waveguide lattice structures synthetically organized using the LISW process. The resulting periodic waveguide structures consist of high index cylindrical cores that are rich in the fluorescent dye, with improved quality in the waveguide lattice structure as well as light collection properties. As solar cell encapsulants, these dye-incorporated waveguide lattice materials play three critical roles in optical functionality: down-converting <400 nm light into the 500–650 nm region, enhancing wide angular collection of visible to near-infrared (vis–NIR) light, and re-emitting portions of light in more directed pathways to the solar cell. Our results show that MWGLs yield the greatest short current density over the angular range (0° to 70°) for three different dye concentration, and that a 0.1 wt % dye concentration leads to the greatest relative enhancements over all structures explored. Our results indicate that in the UV-blue region, down-conversion and confinement of this light within the waveguide structures are the two factors that enhanced energy conversion, and in the vis–NIR region, a combination of down-conversion (of the blue region), confinement of emitted light, and wide-angle light collection of incident light aids in boosting energy conversion. Our findings provide valuable insights into the use of light-responsive waveguide lattices as a potential light management method to expand solar cell performance. By converting UV-A radiation into visible light and collecting more light over a wide incident angular range, the waveguide lattices could significantly enhance the efficiency and reliability of solar cells for both land-based and space applications.

## Experimental Section

### Materials

The photoreactive components used in this study were a high refractive index Norland Optical Adhesive 65 (NOA 65), purchased from Norland Products Inc., and low refractive index epoxide terminated PDMS oligomer, from Sigma-Aldrich, and fluorescein *o*,*o*′-dimethacrylate, also purchased from Sigma-Aldrich. The free-radical photoinitiator camphorquinone (CQ), from Sigma-Aldrich, and cationic initiator (4-octyloxyphenyl)phenyliodium hexafluoroantimonate (OPPI) from Hampford Research, Inc., were employed. All chemicals were used as received.

### Preparation of Photopolymerizable Media

This study prepared two different formulations: pure NOA65 and a binary blend of NOA65 and PDMS. For each formulation, the relative weight fractions of 2.5 and 1.5 wt % were employed for CQ and OPPI, respectively (percentage of total mass weight). The selected binary photoreactive blend had a composition of 20/80 (wt %/wt %) of PDMS/NOA65 for all formulations. The dye concentrations employed herein were 0, 0.1, and 0.5 wt %. Samples prepared from precursors containing 0 wt % dye served as control for the purpose of making comparisons. A mask pattern consisting of 40 μm apertures arranged in a square array of 200 μm interspacing was used to produce all waveguide lattices. Light was provided by light-emitting diodes (LEDs), emitting blue light at a peak wavelength of 470 nm, corresponding to the maximum absorption peak of the free-radical initiator of CQ.

### Fabrication of Thin Films

The photoreactive precursors were measured, added to a vial, and wrapped with aluminum foil. The mixture was then mixed with the assistance of a magnetic stir bar and kept under dark conditions for 24 h to form a homogeneously mixed resin prior to use. The blend was injected into a Teflon ring (1.8 cm in diameter) mounted over a thin glass substrate to a height of 3 mm and placed over the center of the optical mask, which was overlaid at the center of the confocal region of LED light sources. The LED light then passes through the mask to generate a vertical array or two arrays of ±25° slant-oriented microscale optical beams (relative to the surface normal), which propagate through the blends to induce the formation of the waveguide lattices, as done previously.^20^ As control samples, uniformly cured NOA65 films were prepared under irradiation with a single, normally incident LED beam, without the use of a photomask.

### Refractive Index Measurements

Refractive index values for photocured formulations and homogeneous blends were measured using an Abbe refractometer (Atago, NAR-1T SOLID).

### Fluorescence Emission

Fluorescence emission spectra were acquired by exciting the fluorophore molecule at the maximum absorption peak of 480 nm. The wavelengths of fluorescence emission collection were selected in the range of 495 and 650 nm for all samples. A hand-held UV dark lamp was used to visually observe the fluorescence of the samples.

### Optical Characterization

A Zeiss Axioscope equipped with an Axiocam 105 color camera, operated by Zeiss imaging software, was used to capture optical images of waveguide lattices. The transverse spatial intensity profile of incandescent light from a QTH source transmitted through photocured samples was captured with a charge-coupled device (CCD) camera (Dataray Inc.), using an optics setup described previously.^22,23,29−31^

### Solar Cell Measurements

A planar multicrystalline silicon screen-printed solar cell (15 cm × 5 cm × 0.5 mm), with a measured short circuit density of 35.5 mA/cm^2^, was used in the experiments. The photocured samples were laminated onto the solar cell (15 cm, 5 cm, 0.5 mm), which was first primed with a 0.12 mm layer of PDMS (Sylgard). Current density–voltage (*J*–*V*) curves of the encapsulated solar cell were collected under solar simulated irradiation (AM 1.5 G). Angle-resolved measurements were performed as described in our previous work.^20^

## Results and Discussion

### Synthesis of Fluorescent Waveguide Lattices

**Figure 1:**
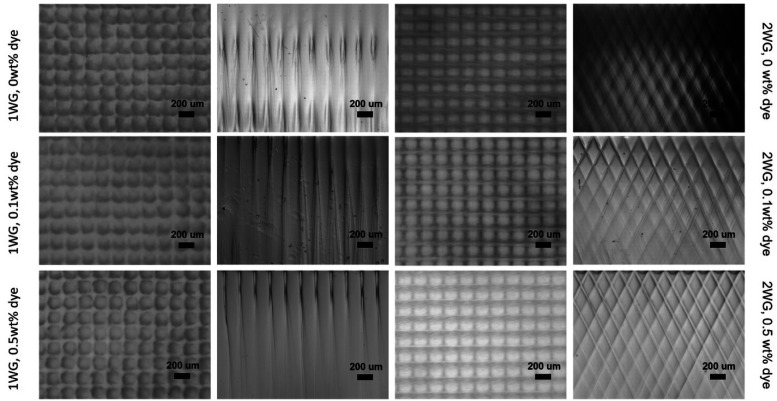
Optical microscopy images of waveguide lattices with 0° and ±25° orientation angles. The structures are fabricated with different concentrations of dye in the precursor mixtures. Columns 1 and 3 show images of the top face of the polymer films, namely, the side from which light enters the film. Columns 2 and 4 show sample cross sections. The scale bar is 200 μm.

We found that including dye in the photoreactive formulations resulted in waveguide cores with more distinct and clear cross sections. The optical images in Figure 1 show the entrance face and cross-section of single waveguide lattice (hereon referred to as WG) films and multiple (two) intersecting waveguide lattice (hereon referred to as 2WG) films (oriented at ±25°) with three different dye concentrations (0, 0.1, 0.5 wt %). The surfaces and cross sections of the lattice and 2WG structures reveal the periodicity and uniformity of the waveguides in their respective lattice and specifically the two intersecting lattices of the 2WG structure. Large scale uniformity in the structure is crucial for their optical properties, particularly for large scale deployment of the coatings over solar cells. The clear and distinct waveguide cores resulting from incorporating dye into the formulations may lead to better transmission of light along the intended direction of the waveguides, ultimately improving performance in certain applications. Cross-sectional images reveal that the waveguide cores produced with dye-incorporated formulations are also clearer and more distinct along their lengths across the film thickness, as indicated by their continuous lateral fiber shape along their length. We found that a dye concentration of 0.1 wt % was optimal for producing the clearest waveguide cores in the case of the single WG lattice (1WG), while for the 2WG structure, a continuous improvement in the quality of the structure was observed with increasing dye concentration, with 0.5 wt % yielding the clearest structure throughout the depth of the film. The benefits of the dye in providing improved contrast are particularly noticeable in the 2WG structures, as we observed improved structure with increased weight fraction of dye. Although the waveguide core diameters in all structures do become divergent and increase in diameter over their length, their consistent diameters at the top of the film for more than ∼1 mm is sufficient to establish the transmission of collected light along the intended direction of the waveguides.^20^ It is quite beneficial that the incorporation of a dye-tagged monomer in the formulation, which copolymerizes with the high-index polymer, contributes to the quality of the structure. The dye may absorb light within the spectral region of the blue LED employed in the synthesis, which could help to absorb light that may stray or scatter into the dark regions, thereby providing better contrast between irradiation and nonirradiation regions. In summary, the optical microscopy images in Figure 1 demonstrate that both 1WG and 2WG structures can be successfully produced from formulations with a dye-tagged monomer, resulting in structures that appear visually of higher quality. These results have implications for the performance of the structures particularly in the field of solar cells, where large scale uniformity in the structure is crucial for their optical properties.

### Fluorescence Emission Properties

**Figure 2:**
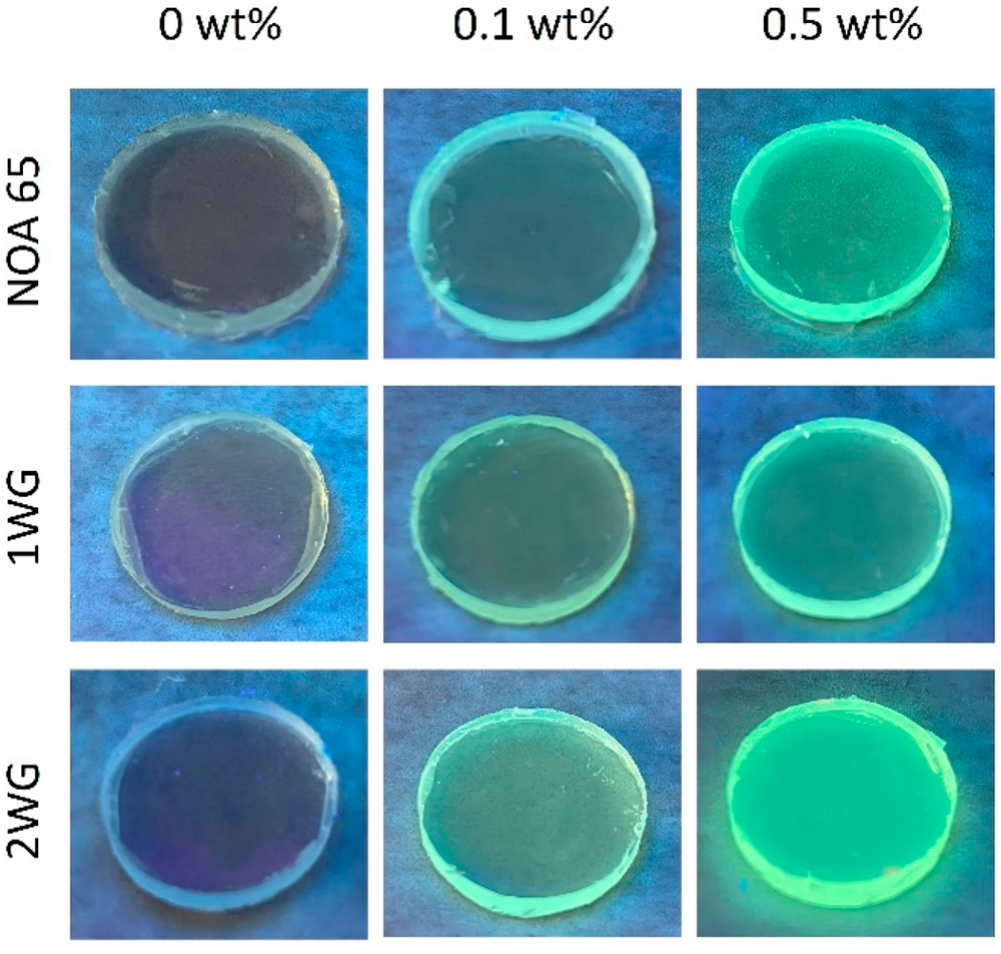
Images of the fluorescence emission of dye under UV irradiation. The dye-incorporated films emit a green-yellow color. The bluish color from the 0 wt % sample is an artifact of the bluish colors of the UV light, confirming lack of fluorescence in this control sample.

Incorporating a fluorescent function of a fluorescein dye via its acrylate-tagged monomer (fluorescein *o*,*o*′-dimethacrylate) is an attractive option due to fluorescein’s very high molar absorptivity (at ∼488 nm), large fluorescence quantum yield (98%), and high photostability, making it an ideal candidate to improve the effective spectrum of light collection for solar cells. We also chose fluorescein dye for its strategic position of its excitation spectrum. To qualitatively examine the emission characteristics of dye in the polymerized thin films and the effect of dye concentration on observable emission, images of their fluorescence were first collected to understand the dye emission in the thin films. Figure 2 shows the images of fluorescence emission from uniform, 1WG, and 2WG structures with 0, 0.1, and 0.5 wt % dye concentrations. First, all dye-containing samples emitted a uniform, bright, yellow-green color. Yet one of the more remarkable observations is that the visual brightness of the sample fluorescence also visually appears to increase from uniform to 1WG to 2WG, namely, with more waveguide lattices in the polymer film. These visual observations are qualitative confirmation of the fluorescence intensities determined from the emission spectra (as discussed later). Samples with 0 wt % were irradiated to provide a visual baseline for comparison and visually confirmed that the polymer had no inherent fluorescence excitation. All samples also exhibited very good transparency.

Figure 2 shows images taken from a non-normally incident perspective view, allowing observation of dye emission that is not normally incident. While the omnidirectional fluorescence emission of the dye may suggest a loss of down-converted light, it is important to note that UV-A light cannot efficiently contribute to the Si solar cell responsiveness. Namely, the efficiency of creating an electron–hole pair from UV-A photons is lower than that of photons with energies closer to the Si bandgap energy, resulting in a higher likelihood of energy dissipation as heat. Therefore, while a significant portion of the emitted photons may not reach the solar cell, any portion of the down-converted photon flux that is collected by the underlying solar cell can provide a nominal increase in energy conversion. This is because the UV-A light would otherwise not be collected at all, owing to the lower efficiency of converting UV-A photons to electron–hole pairs in the Si solar cell.

**Figure 3:**
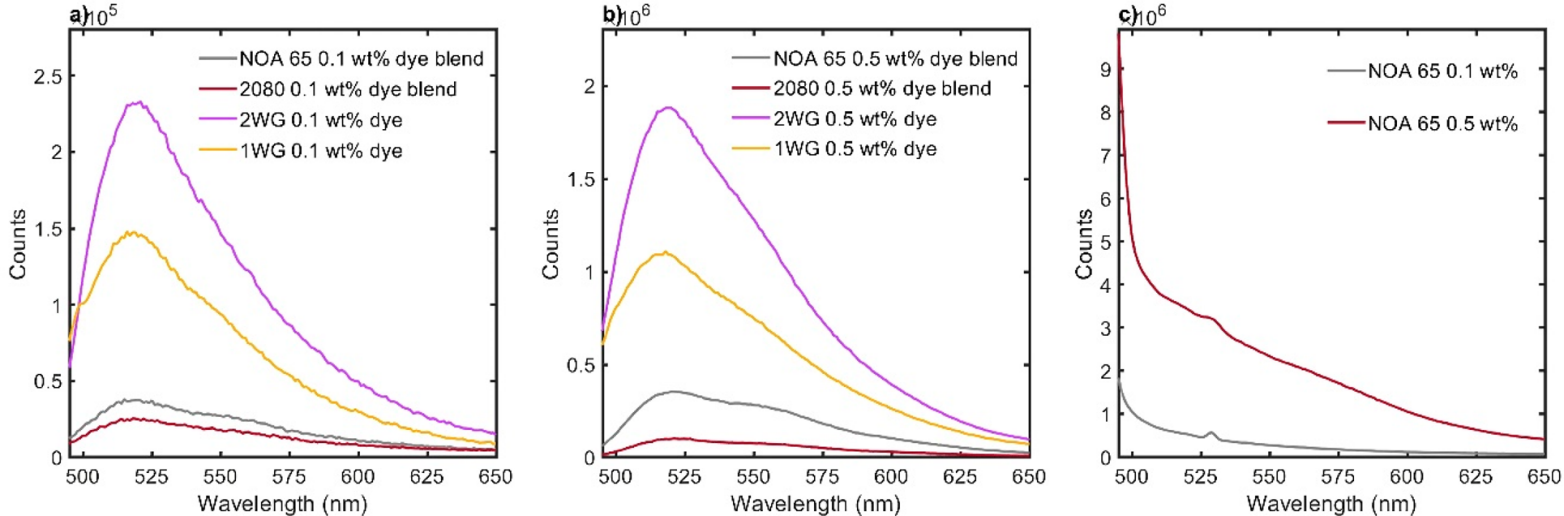
Fluorescence emission spectra of photopolymer blends and waveguide lattice structures. The dye was excited at 470 nm, and the emission spectra were collected with a maximum intensity at ∼511 nm wavelength, with slight variation for all samples. The samples examined include photocurable resins (in liquid form) with NOA65 only and a binary mixture (20 wt % PDMS, 80 wt % NOA65), cured NOA65 resins, and single waveguide lattice (1WG) and multiwaveguide lattice (2WG) structures with either 0.1 or 0.5 wt % dye. Panel a displays fluorescence emission spectra of all samples with 0.1 wt % dye, while panel b displays fluorescence emission spectra of samples with 0.5 wt % dye. Panel c shows the fluorescence emission spectrum of cured NOA65 resin.

To delineate the effects of the different formulation components and structures, we collected fluorescence spectra from four different cured resins: a uniform resin made from only NOA65, a uniform resin from the 20/80 polymer blend, a WG structure produced from the blend, and a 2WG structure produced from the blend. Figure 3 presents fluorescence emission spectra from films for these four systems. The spectra show robust down-conversion and fluorescence of blue light excitation into the green to yellow region from all materials, regardless of structure or dye concentration. The fluorescence emission increased with more structure in the systems, i.e., uniform to 1WG to 2WG. Additionally, the fluorescence emission increased by an order of magnitude when increasing the dye concentration from 0.1 to 0.5 wt %. Given that the fluorescence measurements were performed in transmission mode, this may indicate the capability of waveguide lattice structures to confine and ensure light leaves the material at (close to) normal incidence, an indicator of a beneficial property of controlling and redirecting light propagation, and that this effect is enhanced with the 2WG structure vs 1WG. Waveguides will indeed collect the dye emission internally and coax light to propagate along their long axis toward the other side of the film. This is even more so the case considering that the waveguide cores are composed of rich fluorescent dye tagged acrylate. This ensures that the majority of the emission occurs inside the waveguide cores to begin with, thereby providing the highest likelihood of dye-emitted light being confined by the waveguides and transmitted along their lengths to the other side of the polymer film. In this sense, the inclusion of two waveguide lattices increases the overall density of waveguides, on a per volume basis, and thus doubles the total excitation volume and thus fraction of light that may be confined to and transmitted by a waveguide. Therefore, the fluorescence spectra provide evidence, at normal incidence, for the beneficial, synergistic, and multifunctional properties of polymer films that incorporate both light-active dye emission and waveguide lattice structure. Increasing the dye content and the complexity of the lattice structure (via increasing the number of lattices) increases the overall dye-emitted flux.

We further considered other observations from the fluorescent spectra. The concentration-dependent effect is expected as the amount of dye available for excitation increases with increasing concentration. However, the emission intensity of 0.5 wt % for uniform films from NOA65 and a 20/80 is reversed from those of uniform samples with 0.1 wt % dye, which could be owing to the quenching of excitation or scattering of emitted light. We also noted that fluorescence emission for uniform samples increased from uncured to cured samples (data not shown), possibly due to greater degrees of cure providing better stability and less dissipation of excitation energy allowing for greater emission counts. This degree of cure may also play a role in the improved emission flux from the 2WG vs the 1WG, as the former has double the irradiation dosage (i.e., two LED sources employed), and thus can achieve both a double the lattices in the polymer film as well as overall greater degree of cure. Blends with polydimethylsiloxane (PDMS) show slightly lower intensity of fluorescence emission than the pure NOA65 films possibly due to energy transfer to PDMS upon excitation or loss of energy during emission to the chemical environment in the presence of PDMS.

### Angle-Resolved Optical Collection and Transmission Properties

**Figure 4:**
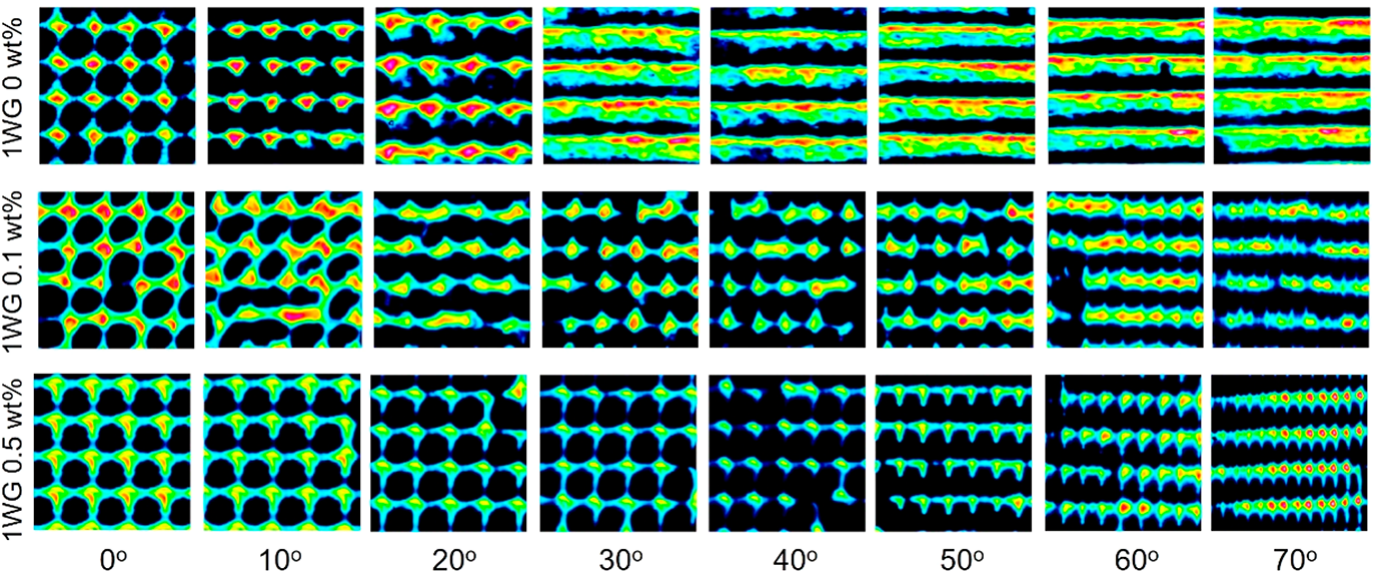
Transverse intensity profiles of transmitted incandescent light through single vertically aligned waveguide lattices over the range of incident angles and three dye concentrations.

Figure 4 shows the transverse intensity profiles of transmitted light through the 1WG structures with different dye concentrations (0, 0.1, 0.5 wt %) as a function of incidence angle. The spot intensities arranged in a periodic array are associated with confinement of light within waveguide cores, indicating efficient collection and transmission of light through optical waveguiding. 1WG structures with no dye collect and transmit light through optical waveguiding within an angular range of 0–30°, as predicted by theoretical calculations based on refractive index differences.^20^ Importantly, incorporating dye into the waveguide lattice structure extends the angular range of light confinement to the entire range examined, from 0 to 70°. The spots in the optical intensity profile are smaller for waveguide lattices produced with 0.5 wt % dye, indicating stronger confinement that may be associated with an increase in refractive index difference between the core and the cladding (see Supporting Information). This increase in refractive index difference indicated by the greater angular collection range can also explain the higher quality structures observed in optical microscopy images (Figure 1), and this greater contrast is associated with greater degrees of phase separation or the increase in refractive index associated with the incorporation of the dye in the formulation (and specifically in the waveguide cores).

**Figure 5:**
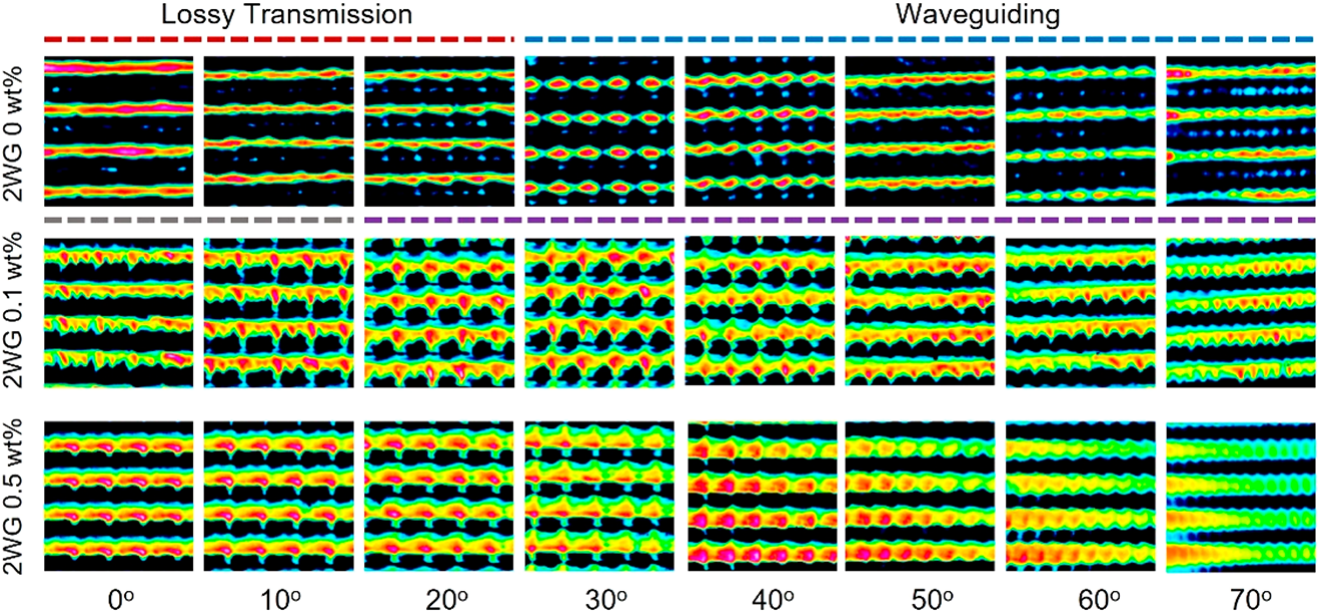
Transverse intensity profiles of transmitted incandescent light through 2WG structures over the range of incident angles and three dye concentrations.

Figure 5 displays the transverse intensity profile of transmitted light through 2WG structures with different dye concentrations (0, 0.1, 0.5 wt %) as a function of the incidence angle. Waveguide structures with no dye content have an angular acceptance window between approximately 30° and 70°, as observed previously.^20^ This particular range is delineated by the spotted nature of the transverse intensity profile, with the approximate circular shape of the intensity spots corresponding to the transmission of light from the waveguide cores. This indicates that light is efficiently collected within the acceptance range of the waveguides at the entrance face of the film and is subsequently transmitted along the long axis of the waveguide cores to the other side of the film. Below this range, the intensity pattern appears smeared, as light can easily pass through the waveguide cores. Our previous work demonstrated that 2WG structures, with each lattice oriented at slant angles with respect to the surface normal period, can collect and transmit light at very wide angles. At low incidence angles, light that is beyond the acceptance range will pass through the waveguides, leading to a more smeared or lamellar pattern in the intensity profile. However, we showed that low angle incident light passing through the waveguides is not detrimental to the light collection process and can even be beneficial due to scattering interactions between the light and the waveguide lattices.

Importantly, Figure 5 also demonstrates that incorporating fluorescent dye into the 2WG structure results in the confinement of light to the waveguide cores across the entire angular range tested (0–70°), as with the 1WG structures. The periodicity of the spots resulting from the organized waveguide cores is evident, though distinct high intensity spots are less observable at lower incident angles. The incorporation of dye offers three possible benefits: First, formulations with dye result in waveguides with higher refractive indices (see Supporting Information), enabling a wider angular range of light to be collected due to the greater refractive index difference between the cores and the cladding. Second, light down-converted from the UV portion of the QTH lamp spectrum may be transmitted through the waveguides despite lossy transmission; thereby sustainable total photon flux leaves the films. Third, since the dye is primarily in the waveguide cores, down-converted light emission also originates from the cores, and the intensity profiles may reveal the expected differences in dye concentration between the waveguide cores and their surroundings. Overall, incorporating dye into the polymer structures enhances the angular acceptance range, providing superior omnidirectional capture of light from normal incidence to an ultrawide angle of 70°.

Notably, larger spot sizes observed in dye-incorporated structures may result from the higher intensity flux of light transmitted through the waveguide cores. Additionally, the spotted nature of the transmitted intensity profiles across the angular range tested provides a visual confirmation of the refractive index profile, specifically the refractive index difference between the core and the cladding. This corroborates the enhanced refractive index difference and wide angular collection properties, which also indicates potentially higher degree of cure and phase separation between the core and cladding.

NOA65 films with different dye concentrations show similar transverse intensity profiles, with a slight increase in intensity observed at mid-incident angles with 0.1 wt % dye (see Figure S2). The uniform intensity profile of the encapsulating provides confirmation of the uniformity of the photocuring process, indicating not significant spatial variations, which is important for subsequent synthesis of waveguide structures, in order to be assured that all waveguides in the lattice have similar structure composition. Some striations observed in the transmission from uniform samples at high incident angles may indicate some degree of convection during polymerization, but the uniformity of the waveguide lattices indicates that such variations do not affect the spatial consistency of the synthesized waveguide lattice structures.

Refractive index measurements did show that higher dye concentration yields a monotonic increase in the average index in both the pure NOA65 resins and the 20/80 polymer blends used to form the waveguide lattices but only by a nominal amount of ∼0.003 (see Supporting Information). This will translate into a slightly wider angular acceptance range of the waveguides, as would be determined by refractive index of high and low index components comprising core-cladding architecture.^20^ The maximum acceptance range of waveguide arrays (without dye) is 30° based on the index of polymer (*n*_NOA65_ = 1.627, *n*_PDMS_ = 1.603). The refractive index difference in NOA65 between 0 and 0.5 wt % dye is so small (Δ*n* = 0.0031) that the difference in angular acceptance range would not be discernible in the transmission experiments. Likewise, the light collection window of slanted waveguide with angular orientation of 25° is determined by first calculation of boundaries (θ_a_) of collection range and rotating the boundaries by addition of waveguide angle,^20^ which gives the collection range of 25° up to 86°, and in the case of the structures produced here in with their slightly higher refractive index, the differences are boundaries that would be 17–90°. Hence, the incorporation of the dye and the capability of these formulations to have a higher refractive index can explain a portion of the extending of the collection window to our lower boundary from 25° to approximately 17°, but it does not completely explain light collection down to normal incidence (i.e., 0°). It is likely that the transmitted light observed below 17° is a combination of lossy transmission through the waveguides which, owing to the higher refractive index, is still able to preserve a greater fraction of light than otherwise possible without the dye or the contribution of light from dye emission. Regardless, the preservation of transmitted light flux through the polymer films, through either collection or dye emission, will ensure greater flux of optical energy to the solar cell, as a means for sustained energy conversion as the incident angle of light varies. In other words, any loss in transmission of incident light may be compensated by dye excitation from the blue to UV-A and its consequent emission in the visible range.

### Solar Cell Performance

**Figure 6:**
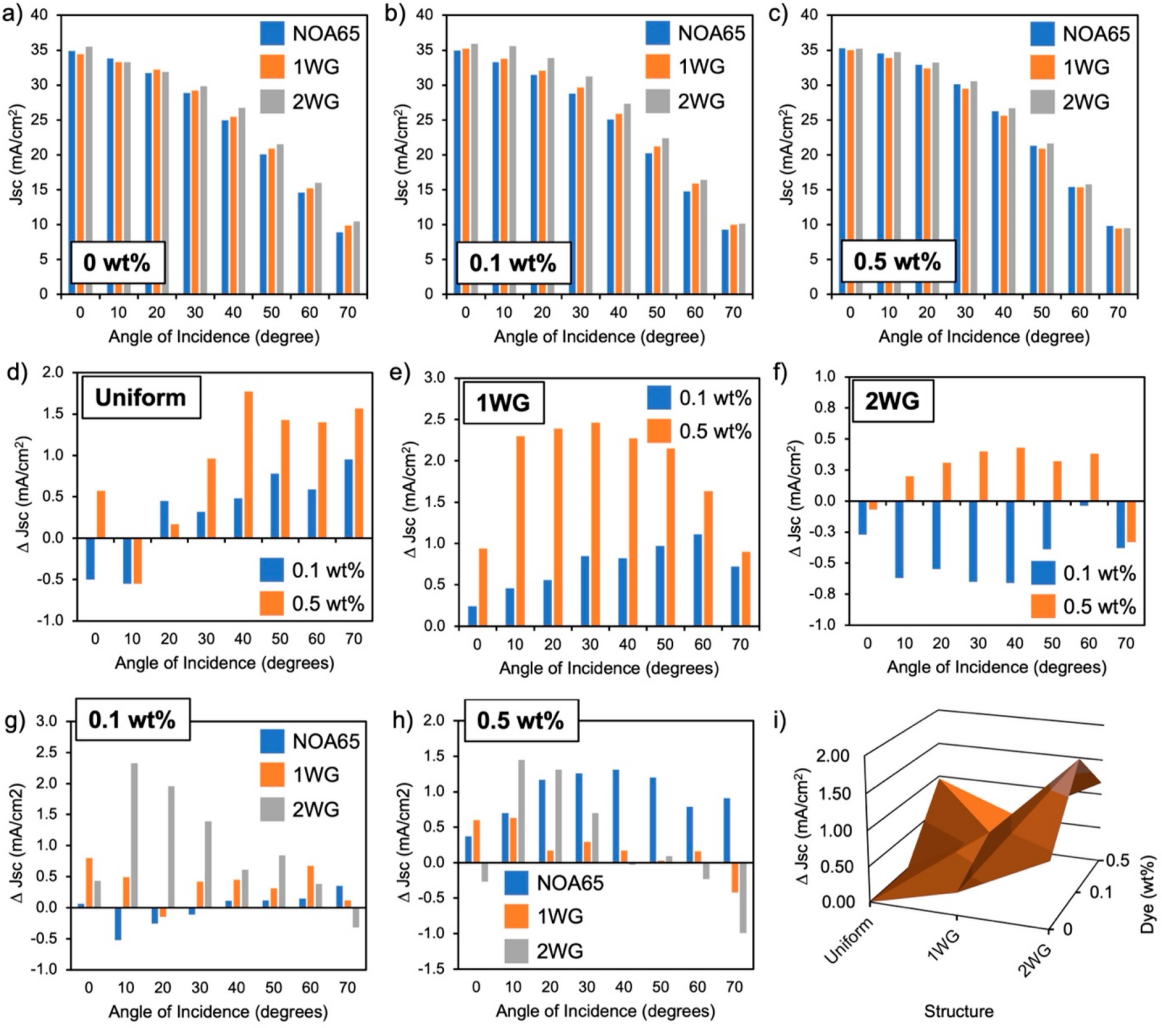
Solar cell performance of encapsulated Si solar cells with dye-incorporated polymer films. (a–c) Short circuit current densities and (d–f) nominal change (Δ*J*_SC_) for thin films with respect to the same structures with no dye. Panels a–c represent thin films with 0, 0.1, and 0.5 wt % dye, respectively, for different structures. Panels d–f represent uniform, 1WG, and 2WG structures, respectively, for different dye concentrations (control is each respective structure with no dye). Panels g and h represent 0.1 and 0.5 wt % dye concentrations, respectively, for different structures concentrations (control is corresponding structure with no dye). Panel i shows a contour plot of the nominal change (Δ*J*_SC_) over all structures and dye concentrations examined.

Figure 6 provides a summary of the current density measurements collected from solar tester experiments on all polymer films and dye concentrations examined (tabulated numerical values provided in Supporting Information). The data provide valuable insights into the enhancement of solar cell current output with different dye concentrations and waveguide structures. The short circuit current densities (*J*_SC_) are summarized bar plots of their values for each angle of incidence examined. All current densities show a characteristic drop in value with increased angle of incidence associated with the shading effect of the front contacts and increase losses from Fresnel reflection at the air–polymer interface. Enhancement provided from either the dye and/or the waveguide structures is observed with nominal increases in *J*_SC_ relative to the control, either the uniform encapsulant, the films with no dye, or both. Examining Figure 6a–c, one key observation is that the use of a 2WG structure leads to higher total current output compared to a single waveguide lattice or a uniform encapsulant, especially at higher incident angles, over all dye concentrations. Another important finding is that the addition of dye into the formulation consistently increases short circuit current density for incident angles in the range of 20–60°. However, the differences between dye and non-dye samples level out at 70°, possibly due to the extreme wide-angle nature of the incident light. With a dye concentration of 0.1%, a monotonic increase in current density over the entire angular incidence range is observed in the order of uniform, 1WG, and 2WG structures. Examining the nominal average current densities (average over incident angle) relative to a uniform encapsulant with no dye, current density increases with increase in the number of lattices in the polymer film (i.e., 1WG, then 2WG). Also, a dye concentration of 0.1 wt % is optimal for both 1WG and 2WG structures. It is also evident that the 2WG structure with dye (0.1 and 0.5 wt %) provides the greatest enhancements in the nominal current density (1.87 and 1.17 mA/cm^2^), providing indication of the synergistic and beneficial nature of incorporating both dye composition and waveguide lattice structure in polymer encapsulant films for solar cells.

To gain more insight into the variations and dependencies of solar cell current density on dye concentration and waveguide structures, Figure 6d–h also provides plots of the nominal difference in short circuit current densities (Δ*J*_SC_) using two different controls: relative to a no-dye control while varying structure (Figure 6d–f) and relative to a uniform control while varying dye (Figure 6g,h), allowing for the clear observation and assessment of the enhancements in solar cell performance with respect to dye incorporation and structure. When structure was used as a control, uniform structures performed better with increasing dye concentration (0.5 wt %), particularly at the high angle range (40–70°) (Figure 6d), while 1WG structures provided enhancements over the entire angular range, which peak in the mid-angular range (20–50°) and increase with increasing dye concentration (Figure 6e). 2WG structures with 0.5 wt % dye provided enhancement, whereas at 0.1 wt % only losses were observed (Figure 6f). When dye concentration is fixed and structure is varied, we observed current density increases for 1WG, and 2WG structures at 0.1 wt % dye (Figure 6g) and current density increases for all structures at 0.5 wt %, with uniform encapsulants providing the most consistent enhancement across the angular range, and 1WG and 2WG structures providing enhancements between 0 and 30° (Figure 6h). At the highest dye concentration of 0.5 wt %, both uniform and 2WG structures performed relatively well, especially for the uniform structure over the entire angular range and for the 2WG structure between 10 and 30°.

To gain further perspective on the effects of dye concentration and waveguide structure, we fitted the average current densities (averaged over all incident angles) to a linear regression model of current density = *A*(structure) + *B*(dye concentration) + *C*, yielding values for *A* = 0.5, *B* = 0.7, and *C* = 0.08. Hence, the dye concentration and structure had a positive effect on current density, resulting in an average gain of 0.5 mA/cm^2^ with the dye incorporation, while waveguide structure results in an average gain of 0.7 mA/cm^2^. As these gains are averaged over the entire angular range, it also demonstrates a positive gain in wide-angle light capture, both through dye incorporation and the waveguide lattice.

By examining the isosurface of gain in current density vs both structure and dye concentration (Figure 6i), we observed maximal enhancements in the total nominal increase in current density, particularly with two waveguide structures (2WG), which maximizes light collection via increased number of lattices in the polymer film, and with uniform structure with maximal dye concentration (0.5 wt %), which maximizes down-conversion. In other words, it is generally better to have more lattices in the encapsulant or dye. There appears to be a strong interaction between dye concentration and waveguide structure such that a lack of structure requires more dye to sustain a greater increase in current density, and likewise, less dye requires more structure to sustain a similar level of enhancement to the current density. The positive slope along the diagonal from uniform with no dye to 2WG structures with 0.5 wt % dye shows a synergistic effect between the dye component and the waveguide structure, indicating that they interact with one another in a way that positively affects the current density. However, saddle point between the two extremes of maximal dye and maximal waveguide structure indicates that a balance between waveguide structure and dye concentration does not provide as much enhancement as the extremes (i.e., more dye and less lattices, or more lattices and less dye). Further investigation and detailed theoretical simulations could help explain the underlying causes of this trend and are planned in the future.

The trends observed in short-circuit current density with respect to waveguide structures are consistent with our findings on the quality of the structures. Specifically, the highest quality structure (1WG at 0.1 wt %) exhibits the highest current density compared to 1WG at 0 or 0.5 wt %. Additionally, improved quality in the 2WG structure, associated with increased dye concentration, corresponds to increased current density. These results highlight the mutual benefits of dye incorporation: the dye not only enhances the lattice structures’ quality but also facilitates the confinement and transmission of the light it emits. The results also affirm structure–property–performance relationships discussed herein. Further enhancements may also be achieved by expanding the composition to include other light-active components, such as plasmonic nanoparticles on the bottom surface of the encapsulant^32^ (in contact with the solar cell) as well as antireflective coatings on the encapsulant surface, which are subjects of continued study.

We also calculated the power conversion efficiencies (PCEs) of the encapsulated solar cells over a range of dye concentrations and waveguide structures (see Figure S3). We observed some similarities and differences compared to the analysis applied to the short circuit current densities. In the case with no dye component, 1WG structures provided the highest efficiencies over the entire angular range. However, when considering short circuit current densities, there was a consistent advantage in increasing the number of waveguide structures (i.e., uniform, 1WG, and then 2WG). For a dye concentration of 0.1 wt %, the 1WG structures provided maximal PCE, but at very wide incident angles, 2WG structures showed better efficiency. On the other hand, when examining the short circuit current densities, the 2G structure was optimal over the entire angular range. At a dye concentration of 0.5 wt %, the uniform structure provided the best efficiencies. However, when considering the short circuit current densities, the performances were comparable. Overall, the analysis of the PCE values still indicates that the combination of dye incorporation and waveguide structure can enhance solar cell performance. However, the incorporation of the dye has a stronger effect on the conversion efficiency.

## Conclusion

In this study, we demonstrated the benefits of incorporating a fluorescent dye excited in the UV to blue region into polymer thin films used as encapsulants for silicon solar cells. The inclusion of the fluorescent dye improved the quality of waveguide lattices produced, enabled down-conversion of blue to UV light into the visible regime, and increased the overall flux of light transmitted through the polymer films. Our findings also revealed that the incorporation of the dye enhanced the current output from solar cells and synergistically worked with waveguide lattices to provide greater transmission of light to the solar cell, leading to further enhancements in energy conversion and electrical output.

Future research will focus on examining the longer-term stability of the polymer films, exploring other multiwaveguide lattice structures, investigating other dye chemistries, and exploring up-conversion processes in the IR for even wider spectral response. Our approach offers a means to achieve enhanced spectral and angular response in commercial Si solar cells, which is critical for increasing their energy output and advancing sustainable green energy into the grid. In summary, our study presents a promising direction for further research and development of more efficient and sustainable solar energy conversion technologies.

## Supporting Information Available

Optical characterization uniform films and refractive index measurements (PDF)
