# Supplementary material for: Fluorescent Waveguide Lattices for Enhanced Light Harvesting and Solar Cell Performance
Source: ACS Appl Energy Mater. 2023 Jun 9;6(12):6646–55. doi: 10.1021/acsaem.3c00687 (PMC10303442; doi:10.1021/acsaem.3c00687)
Supplement: Supplementary file 1 — ae3c00687_si_001.pdf [file ae3c00687_si_001.pdf]

SUPPORTING INFORMATION

Fluorescent Waveguide Lattices for Enhanced Light Harvesting and Solar Cell Performance

Nannan Ding,[1] Ian D. Hosein[1]*

1. Syracuse University, Department of Biomedical and Chemical Engineering, Syracuse, NY, 13244
***Correspondence: idhosein@syr.edu**

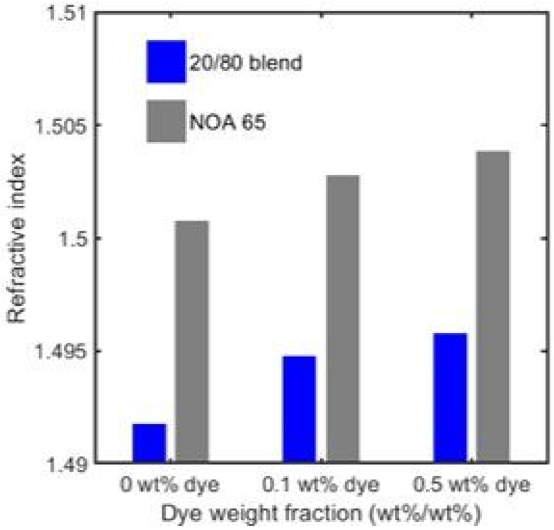

Figure S1. Bar chart of refractive indices of homogeneous binary blends and pure NOA 65 for all concentrations of dye.

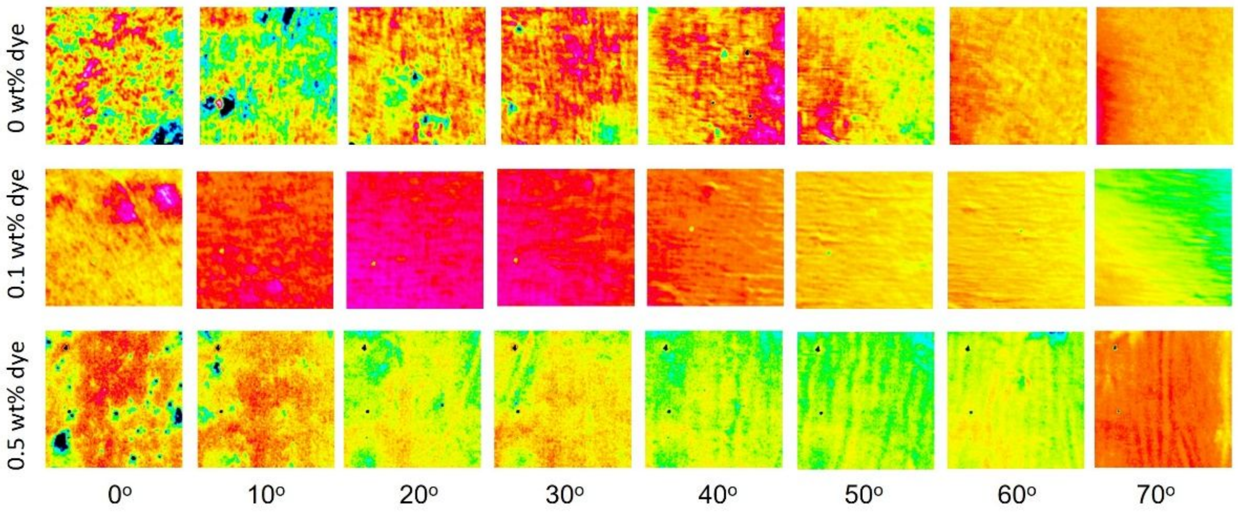

Figure S2. Transverse intensity profiles of transmitted incandescent light through uniformly cured NOA65 over the range of incident angles and three dye concentrations.

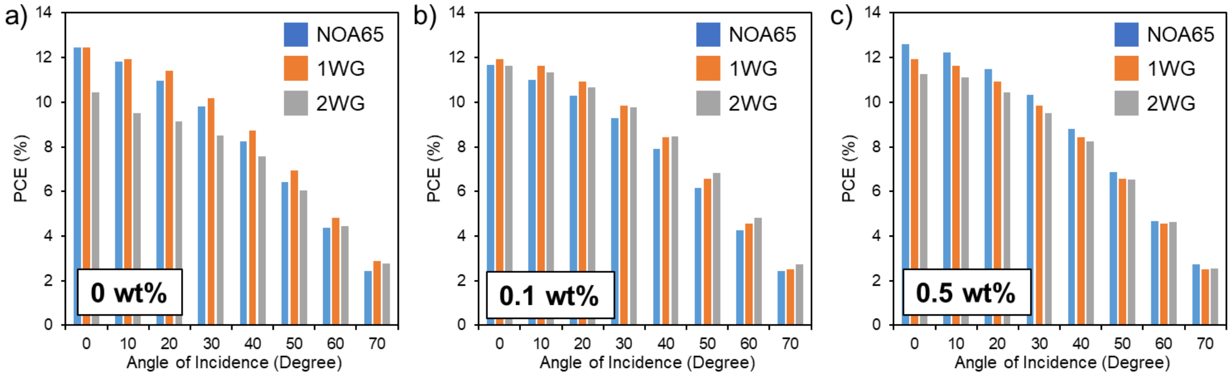

Figure S3. Power conversion efficiencies for Si cells encapsulated with dye-incorporated polymer films with waveguide lattice structures. a) uniform structures, b) 1WG structures, and c) 2WG structures.

Table S1. Summary of J_{sc} vs. angular incidence (0° to 70°) for three different dye concentrations from waveguide lattices.

	AM 1.5 G Solar Irradiation (dye content) (mA/cm²)								
	NOA 65			2080, 1WG			2080, 2WG		
degree	0 %	0.1 %	0. 5 %	0 %	0.1 %	0. 5 %	0 %	0.1 %	0. 5 %
0°	34.91	34.97	35.28	34.41	35.21	35.01	35.48	35.91	35.21
10°	33.81	33.29	34.51	33.26	33.75	33.89	33.26	35.59	34.71
20°	31.74	31.48	32.91	32.19	32.04	32.36	31.91	33.87	33.22
30°	28.88	28.77	30.14	29.20	29.62	29.49	29.84	31.23	30.54
40°	24.96	25.07	26.27	25.44	25.89	25.61	26.73	27.34	26.70
50°	20.09	20.21	21.29	20.87	21.18	20.90	21.52	22.36	21.61
60°	14.62	14.77	15.41	15.21	15.88	15.37	16.02	16.40	15.79
70°	8.91	9.26	9.82	9.86	9.98	9.44	10.48	10.16	9.49
